# Top–down task-specific determinants of multisensory motor reaction time enhancements and sensory switch costs

**DOI:** 10.1007/s00221-020-06014-3

**Published:** 2021-01-30

**Authors:** Ayla Barutchu, Charles Spence

**Affiliations:** Department of Experimental Psychology, University of Oxford, Oxford, OX1 3UD UK

**Keywords:** Multisensory, Audition, Vision, Top–down, Switch cost, Modality-switch effect (MSE)

## Abstract

This study was designed to investigate the complex interplay between multisensory processing, top–down processes related to the task relevance of sensory signals, and sensory switching. Thirty-five adults completed either a speeded detection or a discrimination task using the same auditory and visual stimuli and experimental setup. The stimuli consisted of unisensory and multisensory presentations of the letters ‘b’ and ‘d’. The multisensory stimuli were either congruent (e.g., the grapheme ‘b’ with the phoneme /b/) or incongruent (e.g., the grapheme ‘b’ with the phoneme /d/). In the detection task, the participants had to respond to all of the stimuli as rapidly as possible while, in the discrimination task, they only responded on those trials where one prespecified letter (either ‘b’ or ‘d’) was present. Incongruent multisensory stimuli resulted in faster responses as compared to unisensory stimuli in the detection task. In the discrimination task, only the dual-target congruent stimuli resulted in faster RTs, while the incongruent multisensory stimuli led to slower RTs than to unisensory stimuli; RTs were the slowest when the visual (rather than the auditory) signal was irrelevant, thus suggesting visual dominance. Switch costs were also observed when switching between unisensory target stimuli, while dual-target multisensory stimuli were less likely to be affected by sensory switching. Taken together, these findings suggest that multisensory motor enhancements and sensory switch costs are influenced by top–down modulations determined by task instructions, which can override the influence of prior learnt associations.

**Supplementary Information:**

The online version contains supplementary material available at 10.1007/s00221-020-06014-3.

## Introduction

Events and objects within the environment change continually, not only in terms of their relative spatial location and sensory properties (e.g., a person speaking indoors and outdoors will sound different, yet we have no trouble in recognizing that it is the same person who is speaking), but also in terms of their relevance to a given task or situation. Therefore, the perceptual system, and its ability to coordinate information across the various sensory systems, not only needs to be highly selective and to be able to identify objects accurately but, at the same time, to maintain a high level of flexibility in order to cope with any changes in external bottom-up environmental signals (including noise) as well as with changes in internal top–down task-dependent goals and desires.

For example, when learning to identify the letters of the alphabet, children may orient to the sounds and images of all the letters in order to distinguish them from other characters and sounds in their various forms (e.g., a general interest in letters with specific letter identify irrelevant to the goal at hand). However, while learning how to read a specific word, a teacher may point to, and articulate, each individual letter (in this case, it is the letter’s identity that is goal relevant). Yet, the students would still maintain awareness of other novel signals or events that are somehow irrelevant to the task at hand, despite the presence of conflicting cues, such as the teacher ‘shooshing’ while still pointing at the letter ‘b’ (i.e., noise and incongruent audiovisual pairing informed by past experience). From a young age, the human multisensory perceptual system is capable of dealing with such complexity in the natural environment, even when, as is often the case, there are spatiotemporal overlaps in conflicting multisensory signals. However, there is a limited understanding of how it is that we are able to maintain such high selectivity in multisensory processing, yet be flexible enough to keep up with such changes. Here we focus on task specific selectivity and the influence of congruent and incongruent prior learnt associations on multisensory reaction time (RT) enhancements.

These days, it is widely accepted that multisensory stimulation can facilitate various aspects of human information processing, including perception, learning, memory, and performance. At the same time, however, it can also degrade information processing; thus, providing evidence for the highly selective nature of the multisensory system (e.g., Sinnett et al. 2008). Yet, one does not need much experience with stimuli, nor tasks, to observe large multisensory effects. For example, Miller (1982) demonstrated the multisensory facilitation (i.e., speeding) of RT responses to novel unfamiliar combinations of auditory and visual signals. Such multisensory RT enhancements (in the order of ~ 80 ms) can easily be manipulated by task instructions; RT enhancements are often observed when attention is directed to both senses by having the participants respond to both auditory and visual stimuli (e.g., Giray and Ulrich 1993; Miller 1982, 1991). However, when attention is directed only to audition or to vision by singling out only one sense as being task relevant, then multisensory motor RT gains tend to be smaller (~ 40 ms) (Giray and Ulrich 1993; Miller 1982). Similarly, using the same classic auditory, visual, and audiovisual simple detection paradigm, if participants are instructed to discriminate between the auditory and visual stimuli using different response buttons, then one typically observes slower RTs and reduced awareness for the auditory stimulus when both buttons are to be pressed for multisensory presentations (Sinnett et al. 2008; Spence et al. 2012). This phenomenon is commonly known as the Colavita visual dominance effect (e.g., Hirst et al. 2018; Nava and Pavani 2013; Sinnett et al. 2008; Spence, 2009). Thus, both the selectivity and the flexibility of the multisensory system can easily be modulated by task instructions that alter the task relevance of the sensory signals and, in turn, top–down inputs.

Prior experience can also modulate multisensory processes at both the behavioural and neural levels (e.g., Molholm et al. 2004; Raij et al. 2000). Multisensory RT facilitation is often observed for congruent presentations of well-learnt and easily recognizable stimuli even in the presence of other conflicting signals or sensory distractors (e.g., letters of the alphabet or common objects such as images and sounds of animals) (e.g., Brand-D'Abrescia and Lavie 2008; Chen and Spence 2010, 2013; Downing et al. 2014; Molholm et al. 2004; Raij et al. 2000; Thomas et al. 2017). However, comparable RT enhancements have also been observed using similar audiovisual discrimination tasks with unfamiliar stimulus pairings that do not have any obvious meaningful relationship to one another (e.g., Barutchu et al. 2013; Giard and Peronnet 1999). Furthermore, incongruent presentations of objects (e.g., a barking cat) are often associated with degraded information processing resulting in smaller gains or inhibitory effects (e.g., Chen and Spence 2010, 2013; Molholm et al. 2004; Thomas et al. 2017). Recently, however, Barutchu et al. (2018) demonstrated that even semantically incongruent stimuli (e.g., a barking bird—barking sound + picture of a bird) can give rise to large multisensory enhancements relative to unisensory stimuli. What is more, the benefits were similar in magnitude to those seen for congruent multisensory stimulus presentations (e.g., a barking dog) if the semantic content is irrelevant to the task at hand. At the very least, at the behavioural level, multisensory neural processes can change with response biases and the probabilistic anticipation of multisensory stimuli (Gau and Noppeney 2016; Odgaard et al. 2003; Sarmiento et al. 2016). Indeed, there is plenty of evidence to suggest an anticipatory modulation of neural processing that can be detected prior to stimulus onset (e.g., Corbetta et al. 2000; Ruz and Nobre 2008; Stokes et al. 2009). This may also apply to multisensory neural networks to help maintain a high degree of task-dependent selectivity while, at the same time, remaining flexible enough to keep up with environmental changes and task requirements.

### Sensory switching

The multisensory network needs to be flexible enough not only to deal with distractors and task-irrelevant information, but also to handle unpredictable changes in sensory signals, which tend to have a cost in terms of information processing. It is well-known that sensory- and task-switching can impair (i.e., slow) information processing (e.g., Cohen and Rist 1992; Hunt and Kingstone 2004; Kreutzfeldt et al. 2015; Lukas et al. 2010; Peng et al. 2018; Otto and Mamassian 2012; Shaw et al. 2020; Spence et al. 2001; Liu and Otto 2020). Switching between audition and vision, for example, requires a shift in attention between the senses, which normally takes time. Importantly, however, switch costs are not always comparable in magnitude when switching between unisensory and multisensory stimuli. Therefore, in a typical multisensory detection paradigm, one that involves the random presentation of auditory and visual stimuli, sensory switch costs can interact and potentially inflate multisensory enhancements; multisensory stimulation facilitates RTs while, simultaneously, sensory switching slows down RTs to unisensory stimuli. To date, however, the complex interplay between sensory switching and the task relevance of multisensory signals is unknown.

Here we investigate the effects of target and irrelevant stimuli on multisensory RT enhancements using letters of the alphabet (/b/ and /d/). We were also interested in costs when switching between different types of stimuli (i.e., unisensory vs. multisensory). We used the classic multisensory simple detection paradigm (i.e., a simple RT task) whereby participants were instructed to respond to all stimuli, regardless of the letter(s) that were presented. The prediction was that we would see multisensory enhancements to both congruent and incongruent multisensory stimuli (Barutchu et al. 2018). In the simple discrimination paradigm, a target letter was identified (respond to either ‘b’ and ignore ‘d’, or vice versa—i.e., as in a go/no-go task). Consequently, only one letter at a time was relevant to the task at hand. All other task parameters remained constant. Our prediction was that in the discrimination task, only those stimuli with dual targets (i.e., congruent multisensory letters) would give rise to multisensory RT gains while incongruent multisensory stimuli with a single target component would results in lower gains or possibly even slower RTs as compared to unisensory stimuli (e.g., Miller 1982; Molholm et al. 2004). We also investigated, and demonstrate for the first time, that sensory switch costs (MSE) are partly dependent on the relevance of sensory stimuli—i.e., whether the sensory stimulus is irrelevant (i.e., invalid) or a target.

## Methods

### Participants

A total of 35 participants were recruited and allocated to one of two task conditions: either simple speeded detection or discrimination. The data from four participants had to be excluded due to high error rates (over 2.5 SD above the group mean or over 40% mean error rates). 17 participants were allocated to the simple detection task experiment, one of whom was excluded, leaving 16 participants in the final analyses reported below (age range 19–31 years, *M* age = 25 years 5 months, 11 males and 5 females). The remaining 18 healthy young adults were allocated to the discrimination task, three of whom were excluded leaving 15 participants in the final analysis (ranging between 20 and 32 years of age; *M* age = 23 years 5 months, 7 males and 11 females). According to the literature, multisensory enhancements and switch costs are generally associated with large effect sizes (e.g., Barutchu et al. 2009; Cohen and Rist 1992; Miller 1982). Using G-power, for a mixed design study with two groups, 8 repeating measures, a moderate effect size = 0.4, power = 0.8, and alpha = 0.05, the recommended total sample size is 30 participants for between-group measures analyses and ten participants for repeated measures analyses (Faul et al. 2007). None of the participants reported a prior history of neurological of psychiatric illness. All of the participants either spoke English as a first language or else had started to lean English early in their childhood. The participants were paid £10 for taking part in the study that took approximately one hour to complete.

All of the participants provided informed consent prior to taking part in the study, and all of the procedures were ethically approved and adhered to the guidelines of the Inter Divisional Medical Sciences Research Ethics Committee, University of Oxford.

### Stimuli and procedure

Auditory and visual stimuli consisted of the lowercase letters ‘b’ and ‘d’ (in bold Arial font 72) and their respective phonemes (i.e., /b/ and /d/ enunciated by a mature female) presented for 200 ms. The black letters were presented against a white background in the centre of a 17″ monitor. The auditory stimulus was presented with equal intensity from a pair of external loudspeakers positioned on the sides of the screen, and measured at 75 dB at the participant’s ear (note that this set-up led to the sound appearing to come from the centre of the screen; i.e., from the same apparent location as the visual target). For audiovisual stimuli, the auditory and visual stimuli were presented simultaneously. An oscilloscope was used to confirm the synchronization of the auditory and visual signals (a jitter of less than 1 ms was detected). For each task, the stimuli were presented randomly with equal probability in eight blocks of 240 stimuli. The inter-stimulus interval (ISI) randomly varied between 1250 and 2250 ms in steps of 1 ms. Each block of trials lasted for approximately 7 min. Note that the stimuli and experimental procedures were exactly the same in the detection and discrimination tasks, with only the task instructions changing.

**Fig. 1:**
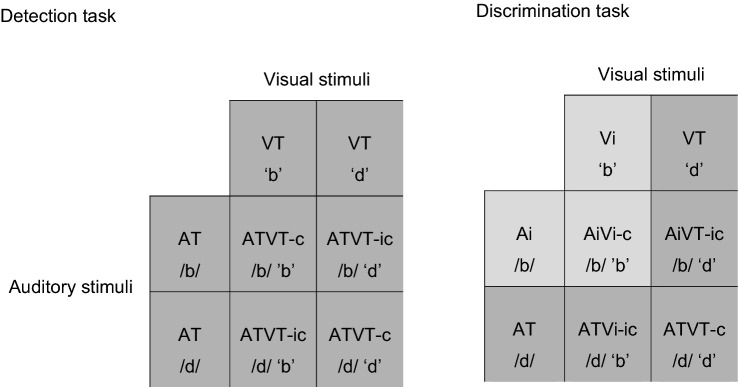
Illustration of the stimuli in the detection and discrimination task. In the detection task, all the stimuli were identified as targets. In the discrimination task, on the other hand, auditory and visual unisensory stimuli were identified as either target (T—shaded in dark grey) or irrelevant (i—shaded in light grey) stimuli (i.e., go (T)/no-go(i), respectively) by letter identity. In the example shown, ‘d’ is the target stimulus and ‘b’ is the irrelevant stimulus (note that, for half of the participants, ‘b’ was the target and ‘d’ the irrelevant stimulus instead). The combination of the four unisensory stimuli results in four multisensory stimuli (8 stimuli in total: 5 targets and 3 irrelevant nontargets). All target stimuli required a motor response (shaded in dark grey). The three irrelevant stimuli were to be ignored (shaded in light grey)

In the simple speeded detection task (i.e., the simple reaction time task), the participants had to respond by pressing a response button to all stimuli as rapidly as possible. There were four stimulus types: auditory (AT), visual (VT), audiovisual congruent (ATVT-c), and audiovisual incongruent stimuli (ATVT-ic) stimuli (see Fig. 1). There were approximately 120 stimuli per stimulus and switch condition.

In this discrimination experiment, the participants were instructed to respond either to the letter ‘b’ (i.e., the target letter) and ignore the other letter ‘d’ (irrelevant letter) or vice versa. Therefore, there were a total of eight stimulus conditions, three of which were irrelevant (i) stimuli that did not require a response: auditory irrelevant (Ai), visual irrelevant (Vi), and audiovisual irrelevant (AiVi-c). The remaining five stimuli were all targets (T): auditory target (AT), visual target (VT), auditory irrelevant with a visual target stimulus (AiVT-ic), auditory target with a visual irrelevant stimulus (ATVi-ic), and an audiovisual target stimulus (ATVT-c) (see Fig. 1). The participants were instructed to make a motor RT response when a target stimulus was detected even if coupled with an irrelevant stimulus, and ignore irrelevant stimuli where no target letter was presented (i.e., Ai, Vi, and AiVi-c) (i.e., similar to a go/no-go task but with all the stimuli type presented with equal probability). The identity of the target letter was counterbalanced across participants. There were approximately 30 trials per condition.

The participants were seated in a quiet sound attenuated room approximately 1 m form the computer screen. The experimenter remained in the testing room throughout a brief practice trial to ensure that the participants understood the task instructions (none of the participants needed more than 50 practice trails). The participants were left alone in the testing room throughout the testing session and were allowed to self-pace their breaks as needed. The experimenter returned at the end of the testing session and debriefed the participants about the study and paid for their time.

### Data analyses

Preliminary analyses revealed that switching between the unisensory graphemes or phonemes did not significantly affect response accuracy or RTs. Therefore, responses for ‘b’ and ‘d’ were collapsed. Only the different unisensory and multisensory stimulus types were analysed further.

Overall, accuracy on both the detection and discrimination tasks was very high with error rates averaging below 10% and some conditions violating the assumption of normality (see Table 1). Nonparametric statistics were used to assess differences in error rates across stimulus and switch conditions.

**Table 1:** Mean percentage error rates (+ SD) and mean RTs (± SEM) for all stimuli as a function of the preceding (pre) stimulus in the detection and discrimination tasks

	preAT	preVT	preATVT-c	preATVT-ic	preAiVT-ic	preATVi-ic	preAi	preVi	preAiVi-c
Detection % error									
AT	3.82 ± 2.78*	5.77 ± 4.00	6.04 ± 4.55	4.96 ± 2.96					
VT	6.61 ± 5.77	**6.32 ± 3.54**	6.74 ± 5.49	5.74 ± 3.43					
ATVT-c	7.66 ± 5.69	6.73 ± 3.59*	7.13 ± 3.93*	6.10 ± 3.65					
ATVT-ic	4.20 ± 2.74*	5.46 ± 4.16	5.35 ± 4.25	**5.45 ± 3.80**					
Discrimination % error									
AT	**2.16 ± 2.07**	5.36 ± 5.19	4.22 ± 3.39		4.59 ± 4.75	3.71 ± 3.91	1.88 ± 2.85	2.45 ± 2.90	3.29 ± 3.79
VT	7.68 ± 9.04	**3.73 ± 5.29**	7.417.00		5.61 ± 5.91	5.57 ± 5.50	1.68 ± 2.06	2.71 ± 2.57	5.45 ± 4.56
ATVT-c	1.19 ± 1.52	3.08 ± 2.64	**1.63 ± 1.83**		1.75 ± 2.15	1.071.91	2.00 ± 2.40	1.00 ± 1.80	1.13 ± 2.75
AiVT-ic	5.47 ± 6.13	3.58 ± 3.93	3.68 ± 5.51		**2.94 ± 2.62**	4.74 ± 4.71	4.933.81	3.94 ± 2.73	3.46 ± 3.41
ATVi-ic	3.86 ± 3.58	6.03 ± 5.50	6.10 ± 6.46		5.59 ± 4.12	**3.77 ± 3.62**	1.94 ± 2.36	4.07 ± 5.07	4.52 ± 2.96
*Ai*	*6.00* ± *6.69*	*3.40* ± *3.69*	*2.57* ± 3.04*		*9.67* ± 8.52*	*4.47* ± *6.63*	0.53 ± 1.42*	*3.98* ± *3.30*	*2.21* ± 3.98*
*Vi*	*4.96* ± *4.19*	*7.78* ± *7.68*	*6.29* ± *6.60*		*4.61* ± *6.18*	*4.63* ± *4.84*	*6.80* ± *6.02*	***3.08 ± 4.13***	*2.47* ± *3.36*
*AiVi-c*	*9.47* ± 11.00*	*8.01* ± 8.33*	*8.30* ± 7.43*		*9.97* ± 11.35*	*8.06* ± 6.50*	*4.74* ± *5.51*	*6.47* ± *4.96*	2.76 ± 3.76*
Detection RT									
AT	**367 ± 17.49**	427 ± 21.30	406 ± 22.38	372 ± 18.02					
VT	392 ± 18.94	**349 ± 17.56**	352 ± 17.76	366 ± 18.10					
ATVT-c	381 ± 20.71	346 ± 19.48	**341 ± 17.76**	351 ± 18.32					
ATVT-ic	336 ± 15.04	328 ± 15.64	325 ± 14.00	**322 ± 13.69**					
Discrimination RT									
AT	**513 ± 16.70**	643 ± 22.81	559 ± 21.84		612 ± 25.19	543 ± 17.71	539 ± 18.88	560 ± 19.69	534 ± 18.28
VT	549 ± 17.71	**487 ± 18.79**	529 ± 22.93		513 ± 19.01	559 ± 20.15	517 ± 15.41	514 ± 13.42	535 ± 15.40
ATVT-c	456 ± 14.78	460 ± 18.01	**449 ± 15.53**		457 ± 15.07	464 ± 13.48	446 ± 13.72	454 ± 13.46	449 ± 11.43
AiVT-ic	581 ± 30.18	521 ± 21.75	565 ± 23.86		**502 ± 15.59**	576 ± 25.17	543 ± 15.42	519 ± 16.93	556 ± 18.77
ATVi-ic	576 ± 18.79	688 ± 33.41	576 ± 20.71		647 ± 27.85	**532 ± 15.23**	540 ± 22.44	571 ± 19.65	551 ± 18.23

Error rates for irrelevant stimuli (i.e., errors of commission/false alarms) are highlighted in italic. All other errors are omissions (i.e., misses) for target stimuli. The repeat conditions are presented in bold font

The ‘pre’ refers to the preceding stimulus (e.g., and AT stimulus with a preAT is a repeat condition, while a VT with a preAT is a visual stimulus that switched from audition on the preceding trial)

^*^p < 0.05 for error rates that significantly differed from other stimuli (see text for details)

For each individual, only RTs greater than 100 ms and less than 3 SD below the mean RT were accepted as correct responses and included in the analyses reported below. Note that less than 1% of RTs were rejected based on these exclusion criteria.

The effects of stimulus switching on accuracy and RTs to unisensory and multisensory stimuli were analysed using analysis of variance (ANOVA; see “Results” section for details). All significant interaction effects were followed-up with paired planned contrasts comparing the repeat stimuli against each other and the switch conditions for each stimulus type.

For each individual and stimulus type in the detection and discrimination tasks, cumulative probability functions (CPFs) were also calculated. For each individual, RT probability along the CDF was calculated starting at 0.05 probability in steps of 0.1 probability for the repeat conditions and the switch conditions separately. Note that, for the detection and discrimination tasks, we collapsed across all of the switch conditions for each stimulus type. The classic Miller’s test of the race-model inequality was also calculated for each individual, which makes a very specific prediction that violations of race models can be assumed if the CDF for the multisensory CDF is faster than the unisensory ‘bound’ CDF (i.e., when the probabilities of the unisensory RT are added together) (see Miller 1982, for details; and Innes and Otto 2019; Liu and Otto 2020; Nardini and Mareschal 2015; Otto 2019, for other related approaches for interested readers). Therefore, paired *t* tests were used to run planned contrasts to assess for race violations along the multisensory CDFs and the unisensory bound CDFs in the detection and discrimination task. Race-model predictions only concern the faster end of the RT distribution; therefore, planned contrasts were only applied to probabilities in the 0.05–0.55 range.

The alpha level was set at 0.05 (i.e., *p* < 0.05) and Bonferroni corrections were applied to correct for multiple comparisons where appropriate.

## Results

### Detection RT task

**Fig. 2:**
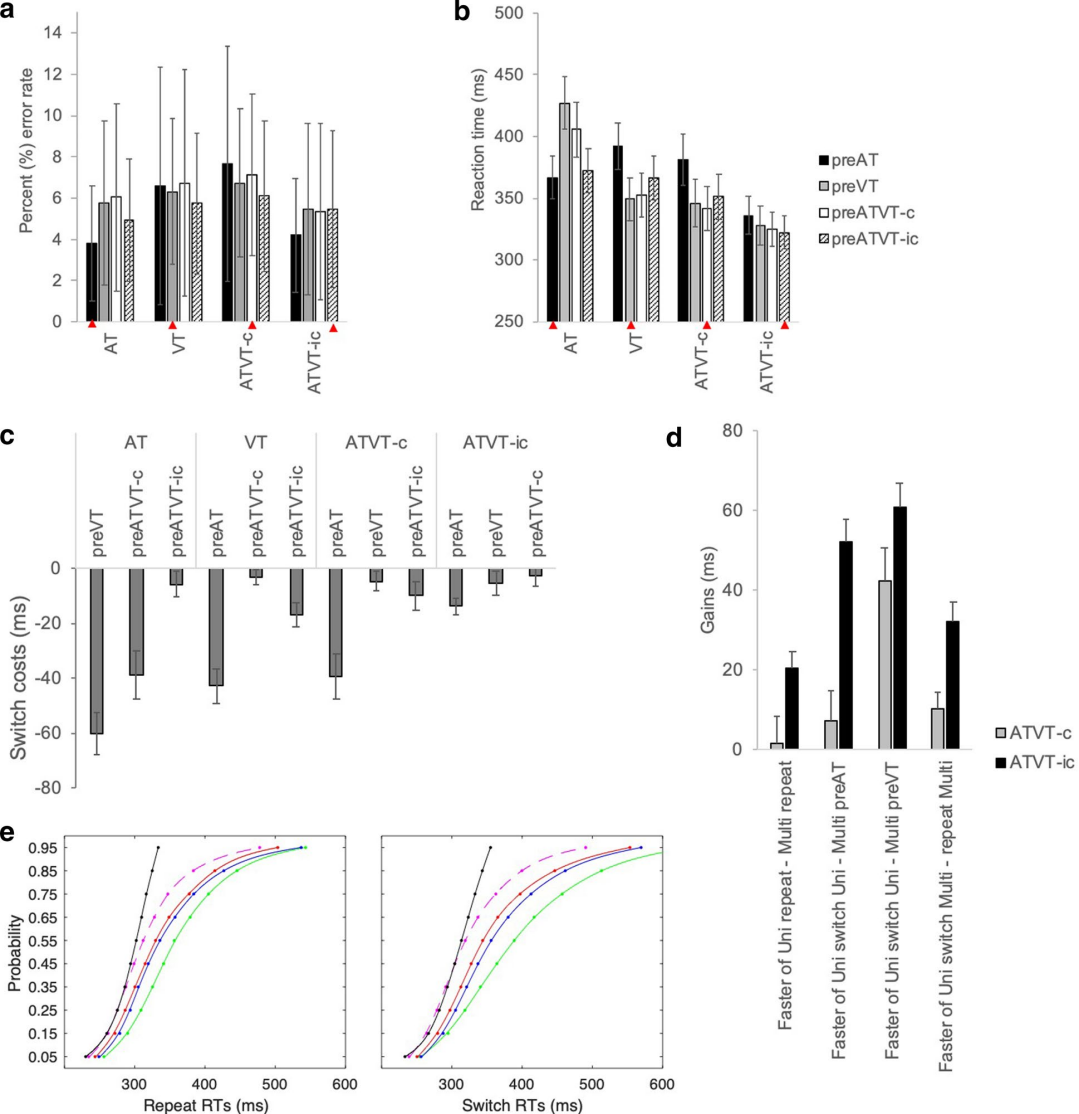
Detection task data. **a** Mean percentage error rates (+ SD), **b** mean RTs (+ SEM) for stimuli and switch conditions. In **a** and **b**, the repeat conditions are marked with a red triangle. Note that the ‘pre’ refers to the preceding stimulus (e.g., an AT stimulus with a preAT is a repeat condition, while a VT with a preAT is a visual stimulus that switched from audition on the preceding trial). **c** Costs in RTs (+ SEM) following a switch. Negative values represent costs in RT speed following a switch. **d** Multisensory RT gain measures (+ SEM) for repeat conditions calculated by finding the difference between the faster of the unisensory repeat and switch conditions and the multisensory ATVT-c and ATVT-ic repeat and switch conditions, respectively. Positive values reflect faster RTs to multisensory stimulation. Note that the *x* axis depicts the formula used to calculate the gains for each condition. **e** Cumulative density functions for AT (green), VT (blue), ATVT-c (red), ATVT-ic (dashed pink), and the bound (AT + VT) CDF stimuli (black) for repeat and switch

For the detection task, error rates averaged below 10% error and violated normality, but were still higher than anticipated for a simple detection task (where less than 5% errors are expected) (see Table 1; Fig. 2a). A Friedman’s test revealed a significant effect, *χ*^*2*^(15) = 36.51, *p* = 0.001. Errors were significantly lower for AT repeat stimuli than for ATVT-c repeat (*p* = 0.03) and ATVT-c stimuli that switched from VT (*p* = 004). Error rates were also significantly lower for ATVT-ic that switched from AT stimuli than repeat ATVT-c stimuli (*p* = 0.02) (see Table 1). Note that any effect of stimulus type and condition is significant but still very small; the average differences across the conditions were less than 4% error and there was much variability across individuals (see Fig. 2a).

RTs were modulated by stimulus type and switch conditions (see Fig. 2b; Tables 1, 2b). A 4 (stimulus type: AT, VT, ATVT-c, and ATVT-ic) × 4 (switch: preAT, preVT, preATVT-c, and preATVT-ic) repeated measures ANOVA revealed a significant main effect of stimulus type, *F*(3,135) = 30.38, *p* < 0.001, *η*^*2*^ = 0.67, and switch condition, *F*(3,135) = 7.41, *p* < 0.001, *η*^*2*^ = 0.33. The interaction between stimulus type and switch condition was also significant, *F*(9,135) = 27.15, *p* < 0.001, *η*^*2*^ = 0.64, which was followed-up by planned contrasts comparing the repeat conditions, and the repeat and switch conditions (see Table 2 for an outline of individual planned pairs and *p*-values). For repeat conditions, only the incongruent stimulus (ATVT-ic) was significantly faster than the unisensory AT and VT stimuli (*p* < 0.001); ATVT-c did not significantly differ from AT and VT stimuli. The unisensory AT and VT stimuli were most affected by switching (see Fig. 2c). AT stimuli significantly slowed down following a switch from VT and ATVT-c (*p* < 0.001 for both), while VT stimuli slowed down following a switch from AT and ATVT-ic stimuli (*p* < 0.003 for both). Responses to both ATVT-c and ATVT-ic slowed down significantly following a switch from an AT stimulus (*p* < 0.001). Overall, significant multisensory RT gains were only observed for the incongruent multisensory stimuli (ATVT-ic) (also see Fig. 2d), and switching to a unisensory auditory stimulus resulted in significant RT costs for all stimuli (see Fig. 2c).

**Table 2:** *p* values for post hoc pairwise comparisons for the detection task comparing repeat conditions and switch conditions for each stimulus

		*p*-values
Repeat vs. repeat		
AT	VT	0.072
AT	ATVT-c	0.03
AT	ATVT-ic	< 0.001*
VT	ATVT-c	0.129
VT	ATVT-ic	< 0.001*
ATVT-c	ATVT-ic	0.047
Repeat vs. switch		
AT repeat	AT preVT	< 0.001*
	AT preATVT-c	< 0.001*
	AT preATVT-ic	0.219
VT repeat	VT preAT	< 0.001*
	VT preATVT-c	0.286
	VT preATVT-ic	0.002*
ATVT-c repeat	ATVT-c preAT	< 0.001*
	ATVT-c preVT	0.205
	ATVT-c preATVT-ic	0.075
ATVT-ic repeat	ATVT-ic preAT	< 0.001*
	ATVT-ic preVT	0.244
	ATVT-ic preATVT-c	0.48

^*^Significant values following Bonferroni corrections for multiple comparisons

To illustrate multisensory gains across the different repeat and switch conditions the difference between the faster of the unisensory conditions and the multisensory congruent and incongruent conditions were calculated, for repeat and switch conditions separately (see Fig. 2d). Contrary to expectations, multisensory gains were greater for incongruent than congruent conditions. Similarly, although CDFs for the multisensory stimuli were shifted to the left of the unisensory CDFs, suggesting faster RTs for multisensory than for unisensory stimuli, this shift was much greater for incongruent than for congruent stimuli (see Fig. 2e). No significant race-model violations were observed for the repeat and switch conditions in the detection task (*p* > 0.01 for all planned comparisons). This null result was surprising given that many previously published studies have demonstrated a significant effect. Elsewhere, we have followed-up on this discrepancy and determined that it may be related to the fact that participants were left alone in the room. Despite the high accuracy, multisensory RTs effects are affected by the absence of the experimenter (see Supplementary information; Barutchu and Spence 2020).

### Discrimination task

**Fig. 3:**
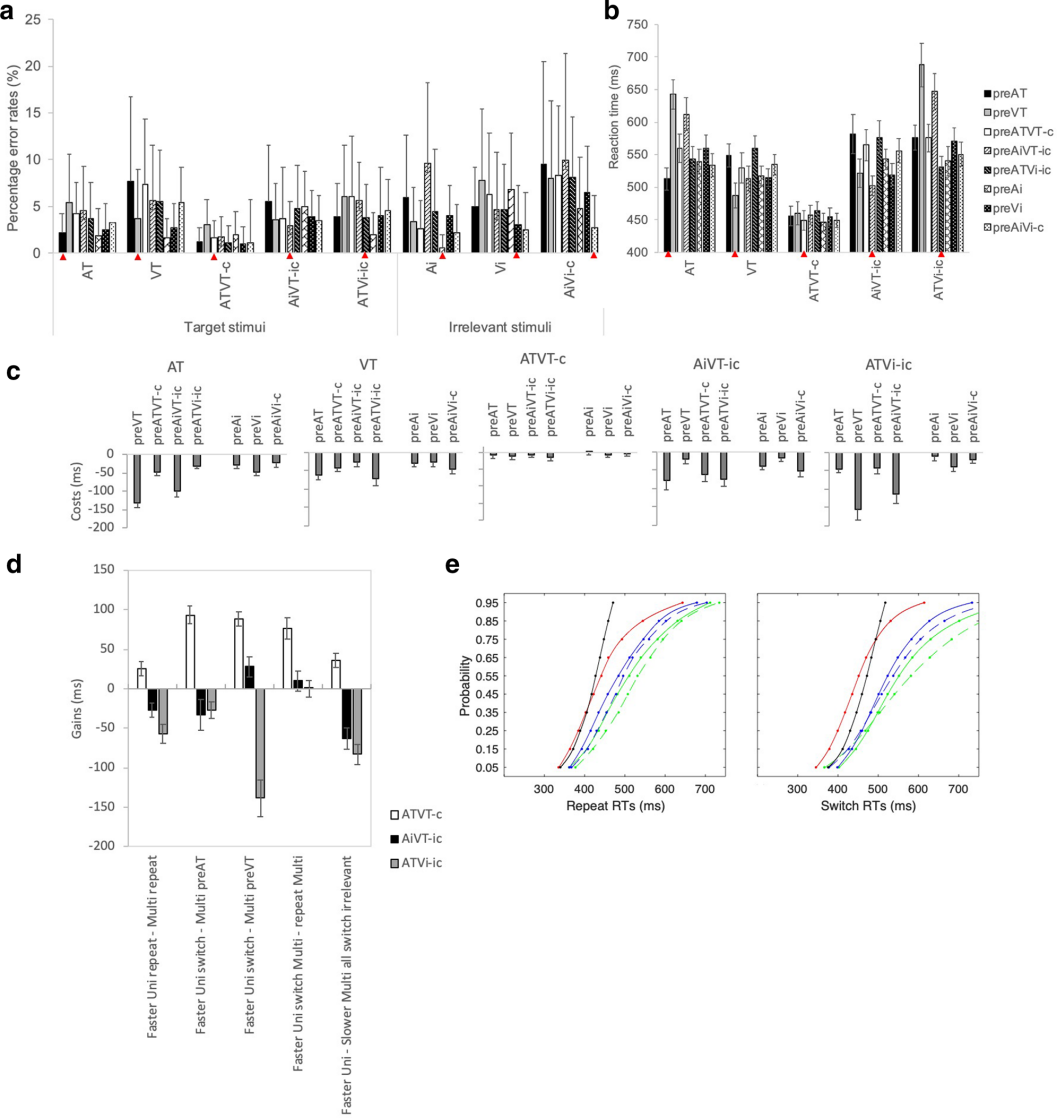
Discrimination task data. **a** Mean percentage error rates (+ SD), **b** mean RTs (+ SEM) for stimuli and switch conditions. In **a** and **b**, all repeat conditions are marked with a red triangle. Note that the ‘pre’ refers to the preceding stimulus (e.g., an AT stimulus with a preAT is a repeat condition, while a VT with a preAT is a visual stimulus that switched from audition on the preceding trial). **c** Costs in RTs following a switch. Negative values represent costs from switching. **d** Multisensory RT gain measures (+ SEM) for repeat conditions calculated by finding the difference between the faster of the unisensory repeat and switch conditions and the multisensory stimuli (ATVT-c, AiVT-ic, and ATVi-ic) repeat and switch conditions, respectively. Positive values reflect gains in RT speed to multisensory stimulation. **e** Cumulative density functions for AT (green), VT (blue), ATVT-c (red), AiVT-ic (dashed blue), ATVi-ic (dashed green) and the bound (AT + VT) CDF stimuli (black) for repeat and switch trials in the discrimination tasks. Note that the AT and VT stimuli were used to compute the bound AT + VT CDF

Error rates for the discrimination task were also very low and violated the assumption of normality. We ran individual Friedman’s test for each stimulus type comparing the different switch conditions, which showed significant effects for AT, *χ*^*2*^(7) = 15.08, *p* = 0.04, Ai, *χ*^*2*^(7) = 33.82, *p* < 0.001, VT, *χ*^*2*^(7) = 17.02, *p* = 0.02, Vi, *χ*^*2*^(7) = 15.15, *p* = 0.03, and AiVi-c, *χ*^*2*^(7) = 23.93, *p* = 0.001. However, after adjusting for multiple pairwise follow-up comparisons, significant differences were only observed for Ai and AiVi-c. For Ai, errors were significantly higher when Ai switched from AiVT-ic, than when Ai repeated (*p* < 0.001), and when Ai switched from AiVi-c (*p* = 0.005), and ATVT-c (*p* = 0.008). For AiVi-c, error rates were significantly higher when switching from the target stimuli, AT (*p* = 0.02), VT (*p* = 0.02), ATVT-c (*p* = 0.03), AiVT-ic (*p* = 0.04), and ATVi-ic (*p* = 0.05) than the repeat AiVi-c stimulus. Error rates did not significantly differ across the switch conditions for ATVT-c, *χ*^*2*^(7) = 12.95, *p* = 0.07, AiVT-ic, *χ*^*2*^(7) = 4.54, *p* = 0.72, and ATVi-ic, *χ*^*2*^(7) = 11.64, *p* = 0.11. We also compared the different stimuli type for repeat conditions and found a borderline effect with no follow-up pairwise significant effects, *χ*^*2*^(7) = 14.04, *p* = 0.05. Any effect of error was very small with a large overlap in variance across conditions (see Fig. 3a), therefore we did not analyse error rates further.

In the discrimination tasks, multisensory RT enhancements were observed only for congruent dual target ATVT-c stimuli (see Fig. 3b). A 5 (stimulus type: AT, VT, ATVT-c, AiVT-ic and ATVi-ic) × 8 (switch: preAT, preVT, preATVT-c, preAiVT-ic, preATVi-ic, preVi, preAi, and preAiVi-c) ANOVA revealed significant main effects of stimulus type, *F*(4,56) = 60.68, *p* < 0.001, *η*^*2*^ = 0.81, and switch condition, *F*(7,98) = 5.17, *p* < 0.001, *η*^*2*^ = 0.27. The follow-up main effects analysis showed that RTs were significantly faster for both the repeat and switch ATVT-c stimuli than for all other stimulus and repeat conditions. There was also a significant interaction between stimulus type and switch condition, *F*(28,392) = 15.82, *p* < 0.001, *η*^*2*^ = 0.53. Follow-up planned contrasts revealed that switch conditions did not affect RTs for ATVT-c stimuli (*p* > 0.1 for all; see Table 3 and Fig. 3c). However, RTs were significantly slower for those stimuli containing a single auditory target (i.e., AT and ATVi-ic) after switching from stimuli with a single visual target (i.e., VT and AiVT-ic) (*p* < 0.01 for all; note that for the AiVT-ic repeat vs. AiVT-ic pre AT comparison *p* = 0.005 which is above criterion after a Bonferroni correction and is likely to be a Type II error). Switching from target stimuli also resulted in greater costs than switching from irrelevant stimuli (see Fig. 3c).

**Table 3:** *p* values for post hoc pairwise planned comparisons for the discrimination task comparing repeat conditions and switch conditions for each stimulus

		*p* values
Repeat vs. repeat		
AT	VT	0.093
AT	ATVT-c	< 0.001*
AT	AiVT-ic	0.35
AT	ATVi-ic	< 0.001*
VT	ATVT-c	0.004
VT	AiVT-ic	0.199
VT	ATVi-ic	0.008
ATVT-c	AiVT-ic	< 0.001*
ATVT-c	ATVi-ic	< 0.001*
AiVT-c	ATVi-ic	0.011
Repeat vs. switch		
AT	AT preVT	< 0.001*
	AT preATVT-c	< 0.001*
	AT preAiVT-ic	< 0.001*
	AT preATVi-ic	0.002
	AT preAi	0.099
	AT preVi	0.001*
	AT preAiVi-c	0.136
VT	VT preAT	< 0.001*
	VT preATVT-c	0.002
	VT preAiVT-ic	0.055
	VT preATVi-ic	0.002
	VT preAi	0.015
	VT preVi	0.034
	VT preAiVi-c	0.001*
ATVT-c	ATVT-c preAT	0.468
	ATVT-c preVT	0.199
	ATVT-c preAiVT-ic	0.275
	ATVT-c preATVi-ic	0.103
	ATVT-c preAi	0.766
	ATVT-c preVi	0.451
	ATVT-c preAiVi-c	0.985
AiVT-ic	AiVT-ic preAT	0.005
	AiVT-ic preVT	0.211
	AiVT-ic preATVT-c	0.008
	AiVT-ic preATVi-ic	0.002
	AiVT-ic preAi	< 0.001*
	AiVT-ic preVi	0.089
	AiVT-ic preAiVi-c	0.004
ATVi-ic	ATVi-ic preAT	0.001*
	ATVi-ic preVT	< 0.001*
	ATVi-ic preATVT-c	0.009
	ATVi-ic preAiVT-ic	< 0.001*
	ATVi-ic preAi	0.526
	ATVi-ic preVi	0.011
	ATVi-ic preAiVi-ic	0.067

^*^Significant values following Bonferroni corrections for multiple comparisons

The CDFs showed that only the ATVT-c CDF shifted to the left of the unisensory CDFs. Planned comparisons using paired *t*-tests revealed significant race-model violations only for the ATVT-c multisensory stimulus up to 0.55 probability in the switch conditions only (*p* < 0.01 for all planned comparisons); the CDFs for the AiVT-ic and VTAi-ic were very similar and overlapped the VT and AT CDFs, respectively (see Fig. 3e).

### Multisensory RT enhancements and switch costs: detection vs. discrimination task

In the detection task, participants had to respond to all letters as rapidly and accurately as possible. In the discrimination task, the participants had to respond to a specific letter, i.e., either ‘b’ or ‘d.’ Given that the discrimination task (what might be considered a go/no-go task) is harder, RTs are slower in the discrimination task (mean RTs ranging from ~ 450 to 690 ms) than in the detection task (mean RTs ranging from ~ 320 to 430 ms). For these additional analyses, multisensory gain and switch cost measures were converted into percentage gains/costs to control for differences in processing speed across the detection and discrimination tasks (for gain measures in milliseconds see Figs. 2d, 3d).

Percentage multisensory gains in mean RTs were calculated from the faster of the mean unisensory RTs for each individual (i.e., for each individual, gain = faster unisensory mean RT—multisensory mean RT). Percentage gain = (gain/faster of unisensory stimulus) × 100. Thus, positive values represent RT gains in response to multisensory stimuli, while negative values represent slower RTs and inhibition to multisensory stimulation relative unisensory stimulation.

Switch costs (in ms) were calculated by subtracting the repeat conditions from the switch conditions. Percentage switch costs = (switch costs in ms/switch RTs) × 100. Note that negative values represent RT costs following a switch.

**Fig. 4:**
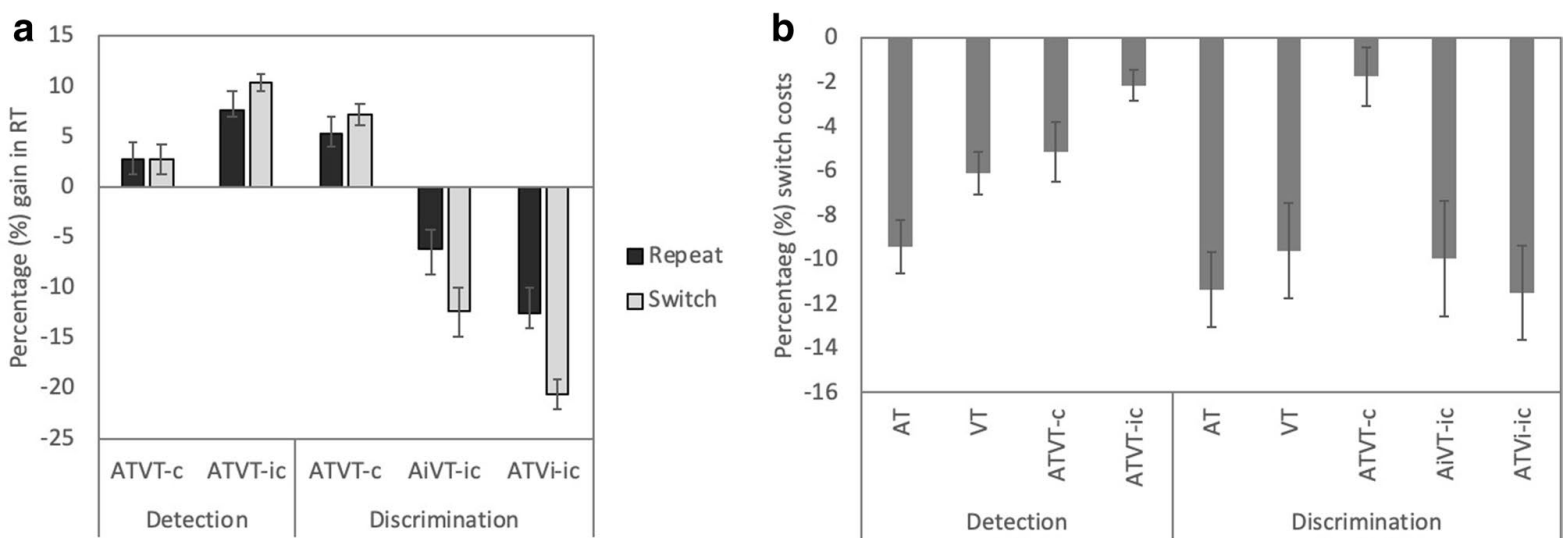
**a** Percentage multisensory gains (+ SEM) in RTs and **b** costs following a switch (+ SEM) in the detection and the discrimination task for repeat and switch conditions for multisensory congruent stimuli (ATVT-c), multisensory incongruent stimuli (ATVT-ic, AiVT-ic, ATVi-ic), and unisensory stimuli (AT and VT)

Correlation analyses revealed that multisensory gain and switch cost measures in ‘milliseconds’ and the converted ‘percentage’ measures correlated very high (*r* > 0.90, *p* < 0.001, for all conditions). Therefore, here we only present the percentage gains and switch costs. Figure 4 shows gains and switch costs, respectively, for both the AiVT-ic and ATVi-ic stimuli in the discrimination task. However, note that for the ANOVA analyses we report here, the two incongruent conditions were collapsed to match the detection task, which by nature has a single incongruent stimulus: congruent and incongruent stimuli were compared statistically.

As can be observed in Fig. 4a, multisensory enhancements to congruent and incongruent stimuli are modulated by task instructions. In the detection task, both congruent and incongruent stimuli led to RT enhancements. On the other hand, in the discrimination task, multisensory enhancements were only observed for congruent letters, while incongruent letters resulted in slower RTs. For the discrimination task, gain measures were collapsed across the two incongruent trials (ATVi-ic and AiVT-ic) to be able to compare percentage gains with the detection task (ATVT-ic) using a 2 (congruent vs. incongruent) × 2 (repeat vs. switch) × 2 (group: detection vs. discrimination) mixed ANOVA. The main effects of stimulus congruence, *F*(1,29) = 40.22, *p* < 0.001, *η*^*2*^ = 0.58, and task type, *F*(1,29) = 47.98, *p* < 0.001, *η*^*2*^ = 0.62, were significant. The three-way interaction between stimulus congruence, switch condition, and task type was also significant, *F*(1,29) = 24.75, *p* < 0.001, *η*^*2*^ = 0.46. Follow-up simple effects analyses revealed that percentage gain measures for congruent and incongruent stimuli differed from each other significantly both within and across tasks (*p* < 0.009 for all). Percentage gain measures were higher for congruent switch stimuli in the discrimination than the detection task (*p* = 0.03). For incongruent stimuli, significant percentage gains were observed in the detection task, but RTs significantly slowed down in the discrimination task for all incongruent trials (*p* < 0.001 for all). Percentage gain measures for repeat and switch trials only differed significantly for incongruent stimuli in the discrimination task (*p* = 0.005). Thus, incongruent multisensory stimuli resulted in significant RT gains in the detection task, while the same incongruent stimuli had an inhibitory effect and slowed RTs in the discrimination task (see Fig. 4a).

The magnitude of switch costs also significantly differed across the two tasks (Fig. 4b). A 4 (stimulus: AT, VT, and multisensory congruent and incongruent stimuli) × 2 (group: detection vs. discrimination) mixed ANOVA was used to assess switch cost differences across the detection and discrimination task groups. Main effects of stimulus type, *F*(3,87) = 13.39, *p* < 0.001, *η*^*2*^ = 0.32, and the interaction between task and stimulus type, *F*(3,87) = 4.56, *p* = 0.005, *η*^*2*^ = 0.14, were significant. For the detection task, switch costs for AT and VT were significantly greater than for the multisensory congruent and incongruent conditions (*p* < 0.03 for all). However, in the discrimination task, switch costs were significantly smaller for the ATVT-c than for the unisensory and incongruent stimuli (*p* < 0.001 for all). Switching had a higher cost for incongruent stimuli in the discrimination than in the detection task.

## Discussion

Here we demonstrate that multisensory enhancements and switch costs are not only modulated by prior learning and experience, but by the task relevance of sensory signals as well. In the discrimination task, multisensory RT enhancements were only observed for congruent letters. Incongruent letters with single targets (i.e., AiVT-ic and ATVi-ic) resulted in slower RTs than the unisensory stimuli. In the detection task, by contrast, the same incongruent stimuli resulted in the highest RT gains and significantly smaller switch costs suggesting that the specific task relevance of the sensory signals modulated multisensory enhancements and switch costs.

Multisensory RT enhancements are partly inflated by sensory switch costs (e.g., Innes and Otto 2019; Liu and Otto 2020; Otto and Mamassian 2017; Shaw et al. 2020). In the present study, we only observed race-model violations in the discrimination task for switch trials suggesting that sensory switching was partly driving the multisensory RT enhancements that were observed. However, it is important to note that the switch costs may not explain multisensory enhancements in their entirety. Switching between the unisensory target stimuli results in slower RTs. However, when switching between unisensory and multisensory stimuli, switch cost are relatively minimal. When we account for the slower RTs and convert RT gains into percentages, we see very small differences in mean RT gains between switch and repeat conditions, and much larger modulations by sensory task relevance. Sensory switch costs are theorised to reflect the time needed for participants to shift their attention between the senses (Kreutzfeldt et al. 2015; Lukas et al. 2010; Spence et al. 2001). With multisensory stimuli, a shift in attention across the senses is not essential as at least one of these sensory signals always repeats; the additional sensory signals seems to have a minimal effect on switch costs if both of the sensory signals are relevant to the task at hand (i.e., with dual target stimuli). Incongruent multisensory stimuli with single targets on the other hand, induce similar switch costs comparable to unisensory target stimuli. We even observed visual dominance (Molholm et al. 2004), typically observed with the Colavita visual dominance effect (Egeth and Sager 1977; Koppen and Spence 2007; Spence et al. 2012), with RTs for auditory targets (i.e., AT and ATVi-ic) in the discrimination task slowing down significantly more following a switch from a visual target than vice versa. In general, costs were also high when switching from a target than an irrelevant visual stimulus. It is assumed that in the letter discrimination task presented here, vision dominated attention and, in turn, led to a greater cost when switching to audition. Thus, in both unisensory and multisensory stimuli, task relevant targets capture attention and, if a shift in attention is required to a target in another sensory modality in consecutive trials, this induces a switch cost. The role of attention is further supported by the fact that switch costs were higher in the discrimination than the detection task for incongruent stimuli. Not only is the discrimination task harder and more likely to engage higher levels of attention, the incongruent stimuli in the discrimination task would have required a shift in attention across targets of different senses, thus resulting is higher switch costs. When attention is taxed by a harder task, the cost of switching attention between the senses is significantly higher for an auditory target following a visual target.

Consistent with prior findings, multisensory enhancements were observed for those audiovisual stimuli with the pre-existing associations (Barutchu et al. 2018; Molholm et al. 2004; Raij et al. 2000; Thomas et al. 2017). Crucially, however, this was only the case when the learned associations were somehow task-relevant. In the discrimination task, the participants only had to respond to one letter and were instructed to ignore the other letter; when both the auditory and visual signals were targets, multisensory enhancements were observed. However, incongruent multisensory stimuli with single targets had the opposite effect, slowing RTs. The RTs were slowed more when the visual signal was irrelevant (ATVi-ic than AiVT-ic when audition was irrelevant) thus suggesting that the visual signal may have dominated and interfered with the processing of the auditory targets in the discrimination task. The observation of such visual dominance is not surprising given that we used letters, and reading is predominately a visual activity. A similar pattern of results has previously been observed with novel stimulus combinations without any specific prior learnt associations (i.e., flashes and tones) (Barutchu et al. 2013) and familiar stimuli like animal exemplars (Molholm et al. 2004). Furthermore, Thomas et al. (2017) used a Stroop-like paradigm to demonstrate that irrelevant semantically congruent visual information can facilitates auditory detection, but interfere with the detection of semantically incongruent auditory stimuli, showing that task irrelevant visual information can dominate not only in multisensory, but auditory tasks too.

Consistent with Barutchu et al. (2018) recent findings, here we also demonstrate that when the same letter stimuli are used in a multisensory detection task, by instructing the participants to respond to all of the stimuli, then multisensory enhancements can be observed for incongruent stimuli. Thus, prior learnt associations can be vetoed and ignored if the association happens to be irrelevant to the task at hand. We propose that the task instructions may change the underlying behavioural and neural processes in anticipation of multisensory stimuli (i.e., prior to stimulus onset). Such preparatory neural modulations prior to stimulus onset have, of course, been observed previously in the visual system (e.g., Corbetta et al. 2000; Ruz and Nobre 2008; Stokes et al. 2009), and extend to the multisensory case as well. Preparatory modulation of the multisensory network may help to explain how the multisensory system is able to maintain both a high level of selectivity and flexibility at the same time. Indeed, previous research has shown that probabilistic expectations can modulate the effects of multisensory congruence in audiovisual illusions such as the McGurk Effect and the ventriloquism effect (Gau and Noppeney 2016; Tong et al. 2020). Here we extend this finding and propose that multisensory enhancements of RTs are also dependent on task specific selectivity and target anticipation. Further research is, though, needed in order to investigate whether such task specific selectivity also applies to other multisensory processes (e.g., multisensory illusions, enhancements of learning and memory, etc.).

In this study, we also observed an unexpected modulation by stimulus congruence in the detection task whereby the expected effect was almost reversed. Compared to the previous studies, the observed magnitudes of the multisensory gains in the detection task for congruent stimuli were much smaller (e.g., Barutchu et al. 2018; Downing et al. 2014); significant RT enhancements were only observed for incongruent stimuli (ATVT-ic), and there were no race-model violations for either repeat or switch conditions in the detection task. It is not that surprising that prior learnt associations, like letters, have an effect on both behavioural and neural processes. Multisensory processes begin modulating learning in early infancy and have been shown to enhance incidental learning in both children and adults (e.g., Bahrick and Lickliter 2000; Barutchu and Spence 2020; Broadbent et al. 2018a, b; 2019; Fifer et al. 2013; Kirkham et al. 2019). There is also a vast literature showing that the pattern of neural network activation for novel stimuli as compared to those with prior learnt associations is different (e.g., Barutchu et al. 2013; Gau and Noppeney 2016; Molholm et al. 2004; Raij et al. 2000). For example, multisensory stimuli with associations common in everyday life (e.g., animals, letters) are more likely to be lateralised to the left superior temporal sulcus (STS—where Wernicke’s language area resides) when compared with novel combinations of audiovisual signals (e.g., Beauchamp et al. 2004; Calvert 2001; Raij et al. 2000; van Atteveldt et al. 2007a, b).

The reversal of the congruence effect appears to be related to another unexpected observation. Accuracy on the detection task was above 90% on average and approached ceiling in some conditions, violating the assumption of normality, suggesting that participants were engaging with the task. Nevertheless, one might well have expected to observe lower error rates on such an easy RT task, thus perhaps implying that participants may not have been fully attending to the task. It is well-known that multisensory processes are often modulated by attention (e.g., Spence and Soto-Faraco 2020; Talsma 2015). Novel stimuli tend to naturally capture attention, therefore, if there is little attention spare, this would explain why multisensory enhancements were higher for incongruent than congruent stimuli. One notable difference between the present and past studies is that the participants were left alone in the experimental testing room in the present study while, in the previous studies, the participants were monitored more closely with EEG or with the experimenter seated in the room (e.g., Barutchu et al. 2018; Downing et al. 2014; Molholm et al. 2004). Separately, we have followed-up on this discrepancy. It turns out that when the experimenter is present in the room, both congruent and incongurent letters result in similar multisensory RT enhancements in the detection task (Barutchu and Spence 2020; also see supplementary information). Interestingly, even when we control and match for accuracy levels, we still observed reduced multisensory gains for congruent stimuli in the alone than monitored condition. The presence of the experimenter is likely to increase motivation, vigilance, and attention; thus, potentially enhancing multisensory processes (e.g., Risko and Kingstone 2011). This finding should be highlighted as it shows how sensitive multisensory effects are too subtle changes in experimental conditions, particularly those that are likely to affect a participant’s state of attention. Future research needs to better distinguish neural processes related to task specific relevance and their interplay with other top–down influences, like attention, prior experience, and semantic congruence.

In the present study, all stimuli were presented with an equal probability to maintain a constant level of stimulus expectation. Inherently, this means that the probabilities of switching between target and irrelevant stimuli are not equal. For example, in the discrimination task, the probability of ATVT-c switching from a single target stimulus (i.e., AT, VT, AiVT-ic, and ATVi-ic) is more likely than vice versa, and the probability of switching from an irrelevant stimulus (i.e., Ai, Vi, and AiVi) is less than switching from a target stimulus. There were also far more presentations of each stimulus type in the detection than the discrimination task, because we maintained the duration across the two tasks constant. Here we controlled for sustained attention and any confounding leaning effects that are potentially introduced with longer task durations across multiple sessions, and only changed the task instructions between the detection and discrimination task. However, future studies need to investigate how the probability of target and irrelevant stimuli effect switch costs and multisensory enhancements.

In conclusion, the results of the present study demonstrate that multisensory RT enhancements are dependent on task relevance, which can, on occasion, override, or veto, prior learnt associations. The task relevance of signals also modulates the effects of sensory switching on multisensory enhancements. Switching between incongruent multisensory stimuli resulted in gains in the detection task, but significantly greater costs in the discrimination task. Future studies are needed to determine the neural underpinning of task relevant multisensory processes and their interplay with other top–down processes such as attention, environmental experience and learning.

## Supplementary Information

Below is the link to the electronic supplementary material.

Supplementary file1 (DOCX 9086 KB)

## Data Availability

Data will be available on request.
