# Supplementary material for: Top–down task-specific determinants of multisensory motor reaction time enhancements and sensory switch costs
Source: Exp Brain Res. 2021 Jan 30;239(3):1021–34. doi: 10.1007/s00221-020-06014-3 (PMC7943519; doi:10.1007/s00221-020-06014-3)
Supplement: Supplementary file 1 — Supplementary file1 (DOCX 9086 KB) [file 221_2020_6014_MOESM1_ESM.docx]

**Supplementary information**

Figure S1 shows the sequential moving averages of RTs for the letter detection task when the experiment was performed under monitored conditions (i.e., with the experimenter seated in the room, *N* = 11 and age-matched to the present experiment, only 6 blocks were completed), and under alone conditions with the experimenter absent throughout the testing phase (as in the present experiment with all 8 blocks completed). All other experiment conditions were identical. Even when accuracy levels are controlled and matched for, greater multisensory RT gains can be seen when participants are monitored then when left alone in the room (see Figure S1 – the red and pink lines showing the multisensory stimuli are much lower than the unisensory stimuli shown in green and blue in the monitored compared to the alone condition). Note that there are too few RTs to calculate moving averages for the discrimination task in the main manuscript.


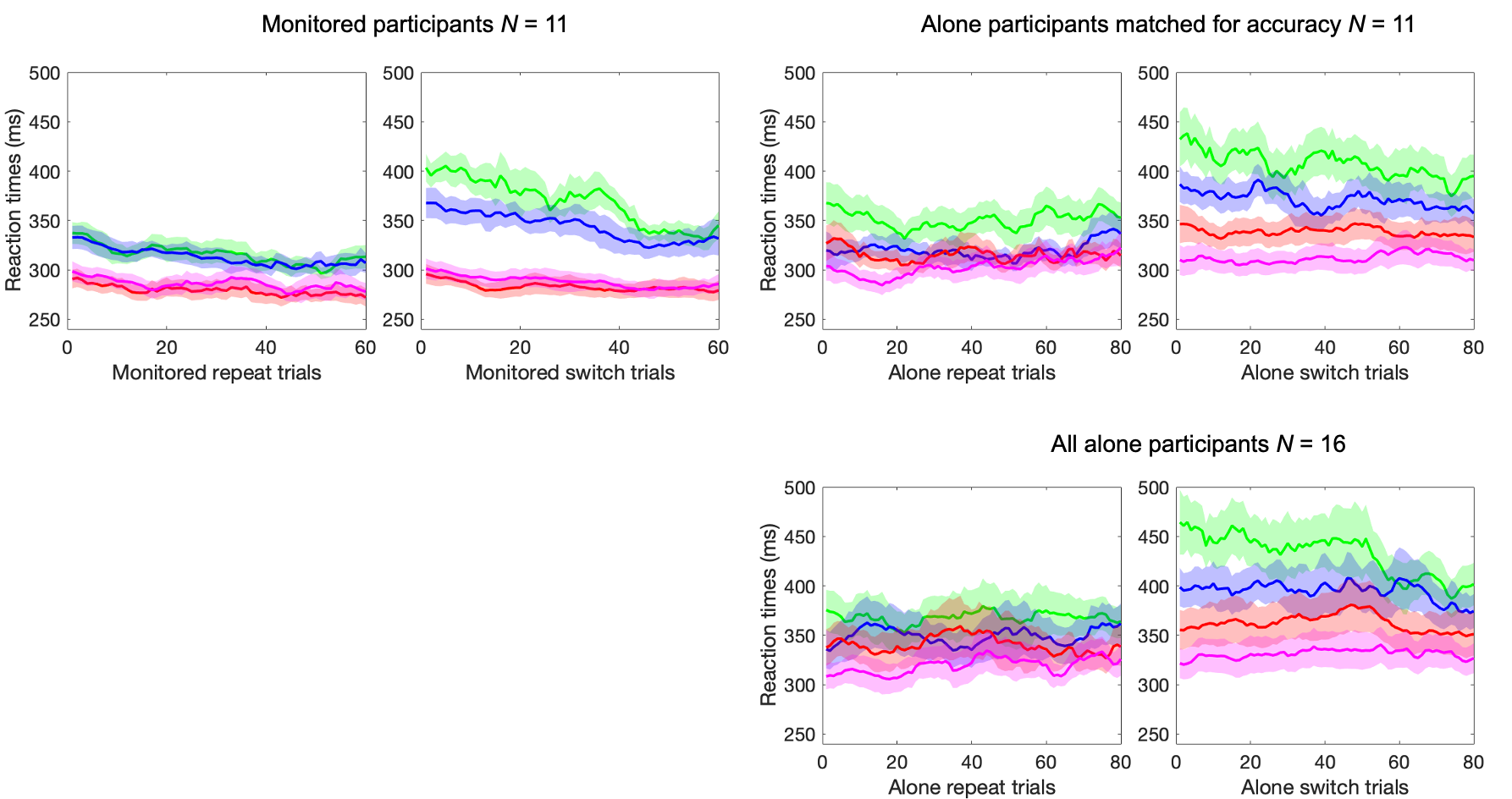


*Figure S1*. Moving averages (+SEM) for the letter switch task: green = auditory stimuli (AT), blue = visual stimuli (VT), red = audiovisual congruent stimuli (ATVT-c), and pink = audiovisual incongruent stimuli (ATVT-ic). Moving averages were calculated by averaging across 10 consecutive trials for each stimulus type moving in steps of 1 across the sequential trials for each stimulus type. Note that the monitored experiment had six blocks rather than eight as in the present study. Therefore, we can plot up to 60 moving average trials in the monitored condition and 80 moving average trials for the alone condition.

Figure S2 further shows that the Cumulative density functions (CDFs) for the multisensory stimuli are also shifted more to the left (i.e., to the faster end of the distribution) than the unisensory conditions in the monitored condition. We also found that congruent multisensory stimuli violated Miller’s predicted race-model inequality up to .55 probability when the participants were monitored (Barutchu & Spence, 2020). For CDFs in the alone conditions refer to main paper.


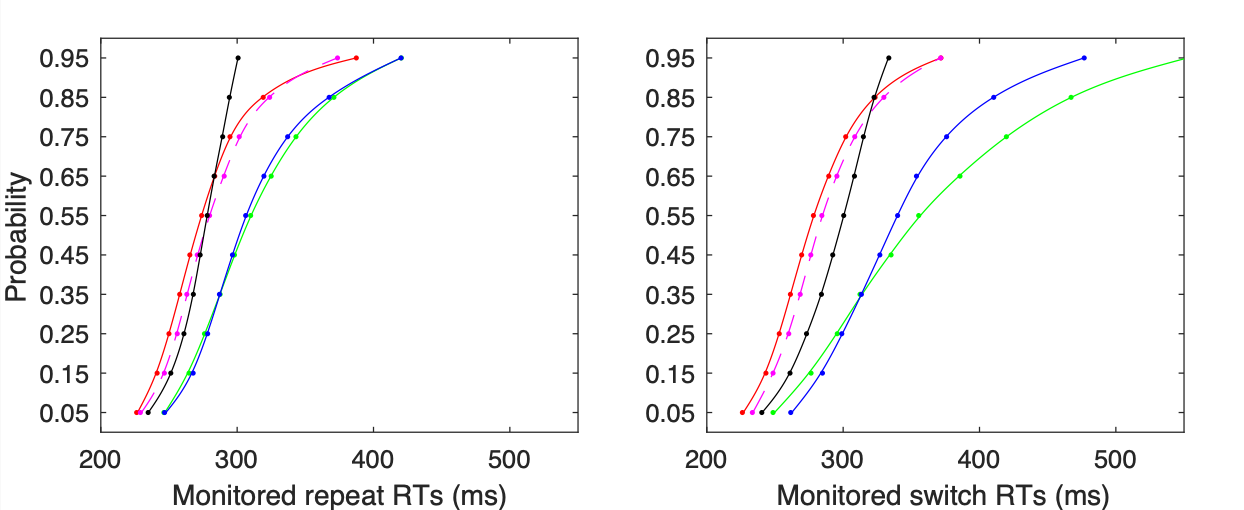


*Figure S2.* Cumulative density functions for the letter detection task under monitored conditions: green = auditory stimuli (AT), blue = visual stimuli (VT), red = audiovisual congruent stimuli (ATVT-c), dashed pink = audiovisual incongruent stimuli (ATVT-ic), black = the bound CDF (i.e., Miller’s race model predicted bound for race-models).
